# Bcl-xL Inhibition Radiosensitizes *PIK3CA/PTEN* Wild-type Triple-negative Breast Cancers with Low Mcl-1 Expression

**DOI:** 10.1158/2767-9764.CRC-22-0024

**Published:** 2022-07-20

**Authors:** Andrea M. Pesch, Benjamin C. Chandler, Anna R. Michmerhuizen, Hannah M. Carter, Nicole H. Hirsh, Kari Wilder-Romans, Meilan Liu, Tanner Ward, Cassandra L. Ritter, Charles A. Nino, Kassidy M. Jungles, Lori J. Pierce, James M. Rae, Corey W. Speers

**Affiliations:** 1 Department of Pharmacology, University of Michigan, Ann Arbor, Michigan.; 2 Department of Radiation Oncology, University of Michigan, Ann Arbor, Michigan.; 3 Rogel Cancer Center, University of Michigan, Ann Arbor, Michigan.; 4 Cellular and Molecular Biology Program, University of Michigan, Ann Arbor, Michigan.; 5 Department of Internal Medicine, University of Michigan, Ann Arbor, Michigan.

## Abstract

Patients with radioresistant breast cancers, including a large percentage of women with triple-negative breast cancer (TNBC), demonstrate limited response to radiation and increased locoregional recurrence; thus, strategies to increase the efficacy of radiation in TNBC are critically needed. We demonstrate that pan Bcl-2 family inhibition [ABT-263, radiation enhancement ratio (rER): 1.52–1.56] or Bcl-xL–specific inhibition (WEHI-539, A-1331852; rER: 1.31–2.00) radiosensitized wild-type *PIK3CA/PTEN* TNBC (MDA-MB-231, CAL-120) but failed to radiosensitize *PIK3CA/PTEN*-mutant TNBC (rER: 0.90–1.07; MDA-MB-468, CAL-51, SUM-159). Specific inhibition of Bcl-2 or Mcl-1 did not induce radiosensitization, regardless of *PIK3CA/PTEN* status (rER: 0.95–1.07). In wild-type *PIK3CA/PTEN* TNBC, pan Bcl-2 family inhibition or Bcl-xL–specific inhibition with radiation led to increased levels of apoptosis (*P* < 0.001) and an increase in cleaved PARP and cleaved caspase 3. CRISPR-mediated *PTEN* knockout in wild-type *PIK3CA/PTEN* MDA-MB-231 and CAL-120 cells induced expression of pAKT/Akt and Mcl-1 and abolished Bcl-xL inhibitor–mediated radiosensitization (rER: 0.94–1.07). Similarly, Mcl-1 overexpression abolished radiosensitization in MDA-MB-231 and CAL-120 cells (rER: 1.02–1.04) but transient *MCL1* knockdown in CAL-51 cells promoted Bcl-xL inhibitor–mediated radiosensitization (rER: 2.35 ± 0.05). *In vivo*, ABT-263 or A-1331852 in combination with radiation decreased tumor growth and increased tumor-tripling time (*P* < 0.0001) in *PIK3CA/PTEN* wild-type TNBC cell line and patient-derived xenografts. Collectively, this study provides the preclinical rationale for early-phase clinical trials testing the safety, tolerability, and efficacy of Bcl-xL inhibition and radiation in women with wild-type *PIK3CA/PTEN* wild-type TNBC at high risk for recurrence.

**Significance:**

This study proposes a novel strategy for the treatment of radioresistant TNBCs using FDA-approved compounds that target apoptosis to improve local disease control in this patient population.

## Introduction

Triple-negative breast cancer (TNBC) is an aggressive subtype of breast cancer with poor rates of locoregional control even after treatment with radiotherapy (RT) (1). TNBC do not express the molecular drivers of tumorgenesis and proliferation such as the estrogen receptor (ER), progesterone receptor, or the HER2, and, consequently, cannot be treated with small molecule and/or targeted therapeutic options. Although radiation is a mainstay in the treatment of breast cancer, patients with TNBC tend to have tumors enriched for intrinsic radioresistance (2) and additional strategies are needed to increase local disease control and reduce the occurrence of regional and distant metastases. Efforts to increase the efficacy of radiotherapy in the treatment of TNBC have primarily focused on targeting DNA synthesis and DNA damage repair, including chemotherapies such as gemcitabine and cisplatin (3) or PARP1 inhibitors such as olaparib and veliparib (4, 5). Although effective radiosensitization agents, these treatments can be associated with extensive normal tissue toxicities that may limit their clinical translatability (6).

In addition to increased DNA damage following radiotherapy, ionizing radiotherapy can lead to increased rates of apoptosis in tumor cells. Apoptosis is a highly regulated pathway of programmed cell death that is controlled by proapoptotic BH3-only proteins (Bid, Bim, Puma, Noxa), proapoptotic effector proteins (Bax, Bak, and Bok), and antiapoptotic BH3 only proteins (Bcl-2 family proteins including Bcl-2, Bcl-xL, Mcl-1, Bcl-w; refs. 7–13). Under normal physiologic conditions, antiapoptotic proteins are bound to effector proteins to inhibit apoptosis (8). Under conditions of cellular stress, the activation of the downstream effector proteins (often Bax/Bak) leads to dimerization and pore formation in the outer mitochondrial membrane, releasing SMAC and cytochrome c; this leads to formation of the apoptosome and the irreversible caspase-mediated cleavage of proteins in the nucleus including PARP1 and, eventually, cell death (14–19).

Careful control of the balance between antiapoptotic and proapoptotic protein signaling cascades is mediated through cellular control of a number of signaling pathways—including the PI3K signaling pathway—which has been shown to directly modulate the expression of Bcl-2 family proteins like Bcl-xL and Mcl-1 (20–23). In breast cancer, it is well known that activating mutations in *PIK3CA*, the gene encoding the catalytic subunit (p110α) of PI3K, occur in almost a quarter of hormone receptor–positive (HR^+^) breast cancers and approximately 10% of TNBC (24). Paradoxically, despite worse outcomes for patients with HR^+^*PIK3CA*-mutant breast cancer, patients with *PIK3CA*-mutant TNBC tend to have higher rates of overall survival compared with patients with subtype-matched tumors that express wild-type *PIK3CA* (24). Similar studies have also studied the effects of the tumor suppressor PTEN, which works in opposition to PI3K signaling in this context (25), where inactivating mutations and loss of PTEN expression lead to higher PI3K pathway activity. Thus, control of cellular apoptosis in breast cancer is partially dependent on the presence or absence of PI3K pathway mutations. Recognizing that patients with TNBC and wild-type *PIK3CA/PTEN* have higher rates of disease recurrence, strategies that are effective in this patient population represent an unmet clinical need (24).

Apoptosis is not the primary mode in which radiotherapy induces cellular death in cancer cells (26), but the clinical development of targeted pharmacologic inhibitors of antiapoptotic proteins has made it increasingly possible to target apoptotic signaling in a variety of cancer types. Inhibition of antiapoptotic proteins as a monotherapy, (specifically Bcl-2, Bcl-xL, and Mcl-1) is a successful cancer treatment strategy for acute myeloid leukemia, chronic lymphocytic leukemia, and small lymphocytic lymphoma (27–30). As a result, efforts are underway to test the effects of inhibiting antiapoptotic proteins in additional cancer types, particularly in combination with other small-molecule treatments such as DNA-damaging agents or compounds that target PI3K/mTOR signaling (20, 31–34). Despite this, few studies have focused on inhibition of antiapoptotic proteins in combination with radiotherapy, and none have specifically examined Bcl-xL inhibition in combination with radiotherapy in the treatment of aggressive breast cancers (35). To that end, a previous study performed in our lab to identify potential targets for radioresistant breast cancers nominated Bcl-2 family inhibition as a potential targeted approach to sensitize radioresistant breast cancers to radiotherapy (36). Thus, we hypothesized that the use of targeted inhibitors against Bcl-2 family proteins in TNBC would be a viable therapeutic strategy for patients with aggressive TNBC without PI3K pathway alterations for whom therapy intensification is needed.

## Materials and Methods

### Cell Culture

Frozen stocks of MDA-MB-231 (RRID:CVCL_0062), CAL-120 (RRID:CVCL_1104), MDA-MB-468 (RRID:CVCL_0419), and CAL-51 (RRID:CVCL_1110) TNBC cells were obtained from ATCC and grown in DMEM (Thermo Fisher Scientific 11965092) supplemented with 10% FBS (Atlanta Biologicals S11650H) and 1% penicillin and/or streptomycin (Thermo Fisher Scientific 15070063). SUM-159 cells (RRID:CVCL_5423) were received courtesy of Steve Ethier and grown in Ham's F-12 media (Thermo Fisher Scientific 11765054) supplemented with 5% FBS, 5 mL of 1 mol/L HEPES (Sigma H3375), 1 μg/mL hydrocortisone (Sigma H4001), 1× antibiotic-antimycotic (Thermo Fisher Scientific 15240062), and 6 μg/mL insulin (Sigma I9278). All cell lines were maintained in a humidified incubator (5% CO_2_), tested for *Mycoplasma* monthly (MycoAlert, Lonza LT07), used for no longer than 6 months of continuous culture, and authenticated at the University of Michigan (Ann Arbor, MI) DNA-sequencing core.

### Gene Expression Knockout

Generation of CRISPR cell lines was performed using the lentiCRISPRv2 plasmid (Addgene 98291, RRID:Addgene_98291). Guides (5′ CACCGAGAGCGTGCAGATAATGACA 3′) targeting *PTEN* were obtained from Integrated DNA Technologies and annealed at 95 degrees and cooled at a rate of 5 degrees per minute. The lentiCRISPRv2 plasmid was digested with BsmB1, purified using the QIAquick Gel Extraction Kit (Qiagen #28706 × 4), and the guide sequences were annealed using T4 DNA Ligase (NEB M0202S). Transient transfection of HEK293T cells (RRID:CVCL_0063) was used to generate lentivirus [1.5 μg PAX2 (Addgene #12260, RRID:Addgene_12260), 0.3 μg MD2.G (Addgene #12259, RRID:Addgene_12259), and 1.5 μg plasmid] in Opti-MEM media. Virus was collected in DMEM containing 30% FBS for 48 hours then centrifuged and filtered (0.45-μm filter). Virus was added to exponentially growing cells for 48 hours with 0.8 μg/mL polybrene after which point hygromycin was used for selection (500 μg/mL). Pooled clones were used for all assays. CRISPR control cells containing a control guide targeting *AAVS1* were also produced for use as Cas9 controls (5′ CACCGGGGGCCACTAGGGACAGGAT 3′).

For transient gene knockdown, si*MCL1* (#L-004501-00), si*AKT1* (#L-003000-00), and a control siRNA (#D-001810-10) were purchased from Dharmacon and used at a final concentration of 25 nmol/L. siRNA were transfected into cells using RNAiMAX (Thermo Fisher Scientific #13378030) in Opti-MEM (Invitrogen #31985-062) with antibiotic-free media. Transfected cells were replated 24 hours after transfection and treated with drug and/or radiation the following day (∼48 hours after transfection). Lysates were harvested from cells 24–48 hours after transfection (as indicated) to assess the efficiency of overexpression and/or knockdown at the protein level. Experimental conditions were similar with transient overexpression of *MCL1* (Origene RC200521) using 1 μg plasmid DNA per well of a 6-well plate.

### Drugs

ABT-263, ABT-199, WEHI-539, and A-1331852 were ordered from MedChemExpress (HY-10087, HY-15531, HY-15607, HY-19741) as 10 mmol/L solutions in DMSO.

### Irradiation

Irradiation was performed using a Kimtron IC-225 at a dose rate of approximately 2 Gy/minute at the University of Michigan Experimental Irradiation Core (225 kVp). Dosimetry is performed semiannually using an ionization chamber connected to an electrometer system that is directly traceable to the National Institute of Standards and Technology calibration. The beam was collimated with a 0.1 mm Cu added filter for cell line irradiation with a half-value-length of 0.51 mm Cu. A Thoraeus filter mm Cu filter (0.4 mm Sn + 0.25 mm Cu) and a half-value-length of 2.29 mm Cu was used for *in vivo* xenograft experiments.

### Western Blot Analysis

Floating and adherent cells were collected and centrifuged to make cell pellets. Pellets were lysed with RIPA buffer (Thermo Fisher Scientific #89901) supplemented with cOmplete Mini protease and phosSTOP inhibitors (Sigma-Aldrich #PHOSS-RO, #CO-RO) and standardized using a BCA protein assay (Thermo Fisher Scientific #23225). Membranes were blocked in 5% milk and primary antibodies were diluted 1:1,000 in 1% milk for use. Quantification of Western blots was performed using Image J software (RRID:SCR_003070).

All primary antibodies were from Cell Signaling Technology and used at a dilution of 1:1,000. Catalog numbers are as follows: cleaved PARP (Cell Signaling Technology 5625, RRID:AB_10699459; 1:1,000), PARP (Cell Signaling Technology 9542, RRID:AB_2160739), cleaved caspase 3 (Cell Signaling Technology 9661, RRID:AB_2341188), caspase 3 (Cell Signaling Technology 14220, RRID:AB_2798429), Cas9 (Cell Signaling Technology 14697, RRID:AB_2750916), Bcl-xL (Cell Signaling Technology 2762, RRID:AB_10694844), Bcl-2 (Cell Signaling Technology 15071, RRID:AB_10694844), Mcl-1 (Cell Signaling Technology 94296, RRID:AB_2722740), pAkt (Ser473; Cell Signaling Technology 4060, RRID:AB_2315049), Akt (Cell Signaling Technology 9272, RRID:AB_329827), PTEN (Cell Signaling Technology 9559, RRID:AB_390810), p110α (Cell Signaling Technology 4249, RRID:AB_2165248), Bax (Cell Signaling Technology 2772, RRID:AB_10695870), and Bak (Cell Signaling Technology 12105, RRID:AB_2716685). The goat anti-rabbit (Cell Signaling Technology 7074, RRID:AB_2099233) and goat anti-mouse (Cell Signaling Technology 7076, RRID:AB_330924) secondary antibodies were used at 1:10,000 for at least 1 hour at room temperature. β-Actin-HRP (Cell Signaling Technology 12262, RRID:AB_2566811) was used as the loading control at a 1:50,000 dilution.

### IC_50_ of Proliferation

Cells were plated in 96-well plates and allowed to adhere overnight. BH3 mimetics were added at various concentrations and, after 72 hours, AlamarBlue (1/10th volume; Thermo Fisher Scientific #DAL1025) was added. Absorbance was measured 3–4 hours after the addition of AlamarBlue with an excitation wavelength of 540 nm and an emission wavelength of 590 nm. Absorbance values were used to calculate normalized growth percentages compared with vehicle (DMSO) controls. Plates were seeded with six technical replicates for each concentration and assays were repeated for three biologically independent replicates. A dose–response curve and IC_50_s were calculated in GraphPad Prism 9.3 (RRID:SCR_002798).

### Clonogenic Survival Assays

Cells were plated at single-cell density (with three technical replicates) in 6-well plates and pretreated for 1 hour with inhibitor. Subsequently, plates were radiated at 0, 2, 4, or 6 Gy. Single-cell colonies were allowed to grow for 1–3 weeks during which time drug-containing media was left on cells without replacement. Cells were fixed with 7:1 methanol and acetic acid and colonies (50+ cells) were visualized with 1% crystal violet staining. Linear-quadratic survival curves were fit to each experimental condition as described previously (37), and radiation enhancement ratios (rER) were calculated as the ratio of the radiation-treated cells divided by the combination treated cells for each treatment group.

### Annexin V Staining

The Annexin V-FLUOS Staining Kit (Roche #11858777) was used to quantify apoptosis and necrosis by flow cytometry. Cells grown in 6-well plates were pretreated for 1 hour with Bcl-2 family inhibitors (ABT-263, ABT-199, WEHI-539, A-1331852) at the indicated concentrations and radiated at 4 Gy. Cells were collected after 48 hours, washed with PBS, and incubated in the dark in 200 μL of binding buffer containing 1 μL of Annexin V-FITC and 1 μL of propidium iodide (PI) for 30 minutes before detection using the Bio-Rad Ze5 flow cytometer. Results were presented as the total percent of apoptotic cells, pooled from *n* = 3 biologically independent experiments, including both early apoptosis (Annexin V+/PI−) and late apoptosis (Annexin V+/PI+).

### *In Vivo* Studies

A total of 2 × 10^6^ MDA-MB-231 cells (13–16 tumors per group) or freshly passaged tumor fragments from patient-derived xenografts (PDX, 11 tumors per group) were orthotopically implanted into the mammary fat pad of 6–8 weeks old CB-17 SCID female mice (RRID:IMSR_CRL:561) sourced from a University of Michigan breeding colony. Tumors were allowed to grow to approximately 80 mm^3^ and randomized before treatment began; mouse weight was an average of 18 g at the start of treatment. Randomization was done to ensure that tumor size was evenly distributed across the four treatment groups, but during the study researchers were not blinded to group assignment. For the MDA-MB-231 xenografts, ABT-263 or A-1331852 were given once a day for 10 days at 25 mg/kg and nine fractions of radiation were given, starting one day after the initiation of drug. For PDX4664 xenografts, drug was started one day prior to radiotherapy, given concurrently with six fractions of radiotherapy, and continued one day after radiotherapy. Tumor size was measured approximately three times per week using a digital caliper. Tumor volume was calculated using the equation *V* = (*L* * *W*^2^) * π/6 (where *V* = volume, *L* = length, *W* = width). Synergistic effects were calculated using the fraction tumor volume (FTV) method as described previously (38, 39).

### Study Approval

The procedures listed above were approved by the Institutional Animal Care and Use Committee at the University of Michigan (Ann Arbor, MI). All patient data were obtained from deidentified online datasets from published clinical trials that were conducted in accordance with recognized ethical guidelines (e.g., Declaration of Helsinki, CIOMS, Belmont Report, U.S. Common Rule) and approved by an Institutional Review Board (40) where written informed consent from all patients was obtained prior to the study.

### Statistical Analysis

Statistical analyses were performed using GraphPad Prism 9.3 (RRID:SCR_002798). One-way ANOVA with Dunnett multiple comparisons test was used for clonogenic survival and Annexin V assays. One-way ANOVA with Dunnett multiple comparisons test at the study endpoint and log-rank (Mantel–Cox) test were used for *in vivo* analyses. (For simplicity, only the statistical comparisons for radiotherapy vs. combination treatment with ABT-263 or A-1331852 are denoted on tumor growth curves.) A *P* value equal to or less than 0.05 was considered significant. A log-rank (Mantel–Cox) test was used to analyze survival data from patients with TNBC that received radiotherapy (sourced from the vandeVijver dataset; ref. 40).

### Data Availability

The data generated in this study are available within the article and its Supplementary Data files, and the data are available upon request. Patient survival data analyzed in this study (Gene Expression Omnibus accession no. GSE30682, ID #200030682) were obtained from publicly available datasets (40).

## Results

### ABT-263, a Nonspecific Bcl-2 Family Inhibitor, Radiosensitizes PIK3CA/PTEN Wild-type TNBCs

**FIGURE 1:**
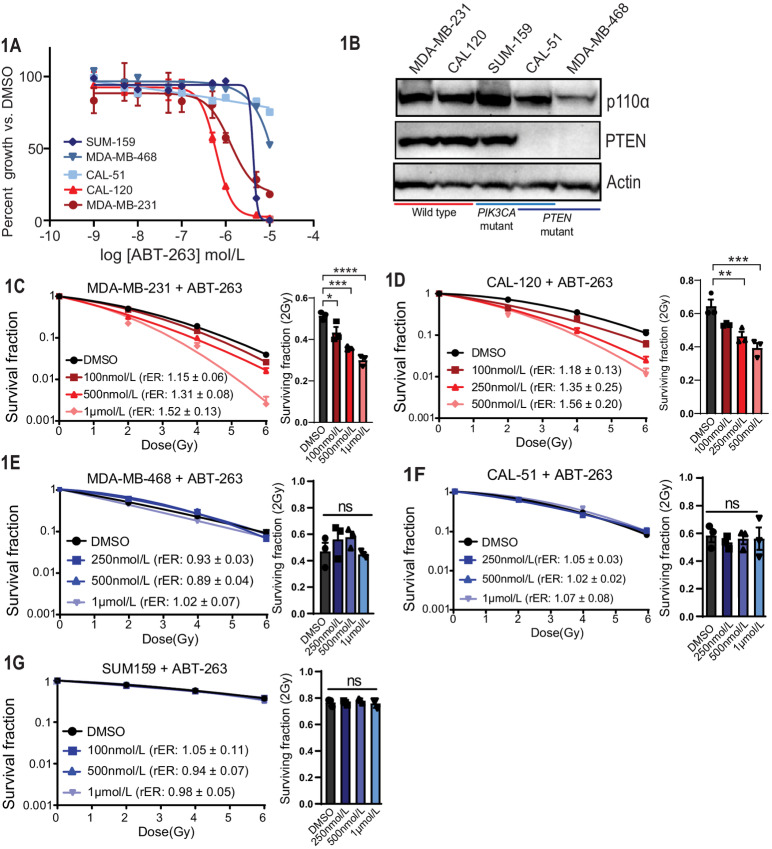
Pan Bcl-2 family inhibition radiosensitizes PIK3CA/PTEN wild-type TNBC. Viability was assessed 72 hours after treatment with ABT-263 in TNBC cell lines (**A**). Expression of PTEN and p110α in TNBC cell lines was assessed by Western blot analysis (**B**). Clonogenic survival assays were used to calculate rERs in *PIK3CA/PTEN* wild-type TNBC (red, **C** and **D**) and *PIK3CA/PTEN*-mutant TNBC (blue, **E–G**). All experiments represent the average of three independent biological replicates and one-way ANOVA with Dunnett *post hoc* test was used to compare the surviving fraction of cells at 2 Gy for each treatment condition in clonogenic survival assays. (ns, not significant; *, *P* < 0.05; **, *P* < 0.01; ***, *P* < 0.001; ****, *P* < 0.0001).

First, we sought to assess the impact of pan Bcl-2 family inhibition on TNBC cell lines *in vitro* (Fig. 1A). Cellular response to the Bcl-2 family pan inhibitor ABT-263 (navitoclax) (41) varied across TNBC cell lines, with IC_50_s less than 1 μmol/L in sensitive cell lines (red) and IC_50_s greater than 5 μmol/L in resistant TNBC cell lines (blue). Consistent with prior studies (33, 42, 43), TNBC cell lines with wild-type *PIK3CA/PTEN* expression (MDA-MB-231, CAL-120) were sensitive to pan Bcl-2 inhibition, while *PIK3CA*-mutant (CAL-51, SUM-159) and *PTEN*-mutant (CAL-51, MDA-MB-468) cells were insensitive (Fig. 1B; Supplementary Tables S1 and S2). Thus, we hypothesized that cell lines with wild-type *PIK3CA/PTEN* would be radiosensitized by ABT-263, while those with mutations in the *PIK3CA/PTEN* pathway would not.

To assess the combined effects of radiation and pan Bcl-2 family inhibition, we performed clonogenic survival assays in *PIK3CA/PTEN* wild-type (MDA-MB-231, CAL-120) and mutant (CAL-51, MDA-MB-468, SUM-159) cell lines. *PIK3CA/PTEN* wild-type cells (Fig. 1C and D) were significantly radiosensitized by ABT-263 (MDA-MB-231 rER 1 μmol/L: 1.52 ± 0.13; CAL-120 rER 500 nmol/L: 1.56 ± 0.20) even at concentrations below the IC_50_ value. In both MDA-MB-231 and CAL-120 cells there was a significant decrease in the surviving fraction of cells after 2 Gy radiotherapy (SF-2Gy) in cells treated with ABT-263 compared with vehicle-treated (DMSO) control cells. Conversely, in PTEN null TNBC cell lines, ABT-263 did not radiosensitize MDA-MB-468 (rER: 1.02 ± 0.07) or CAL-51 cells (rER: 1.07 ± 0.08) and failed to significantly change the SF-2Gy (Fig. 1E and F). Similar results were observed in the *PIK3CA*-mutant cell line (SUM-159 rER 1 μmol/L: 0.98 ± 0.05; Fig. 1G).

### Pan Bcl-2 Family Inhibition Potentiates Radiation-induced Apoptotic Cell Death in *PIK3CA/PTEN* Wild-type TNBC

**FIGURE 2:**
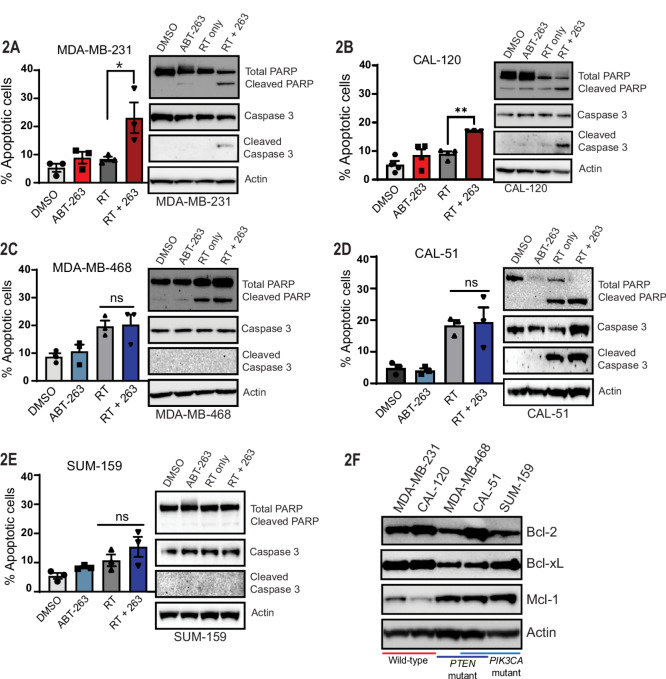
Pan Bcl-2 family inhibition leads to increased apoptosis in PIK3CA/PTEN wild-type TNBC. Apoptosis was assessed by Annexin V/PI flow cytometry in *PIK3CA/PTEN* wild-type TNBC (red, **A** and **B**) and *PIK3CA/PTEN*-mutant TNBC (blue, **C–E**) 48 hours after radiotherapy treatment, and results were pooled for three independent biological replicates. Representative Western blots (from *n* = 3 biological replicates) were used to assess cleaved PARP and cleaved caspase 3 formation 48 hours after combination treatment and the expression of Bcl-2 family proteins (Bcl-2, Bcl-xL, Mcl-1) in each of the TNBC cell lines at baseline (**F**). The concentration for the 1 hour ABT-263 pretreatment was 500 nmol/L in CAL-120 cells and 1 μmol/L in MDA-MB-231, MDA-MB-468, SUM-159, and CAL-51 cells. (ns, not significant; *, *P* < 0.05; **, *P* < 0.01).

Because Bcl-2 proteins are critical components of the cellular antiapoptotic signaling cascade, we hypothesized that ABT-263–mediated radiosensitization of TNBC cell lines was due to an increase in apoptosis following the combined treatment. In our *PIK3CA/PTEN* wild-type TNBC models, ABT-263 + RT (4 Gy) led to a significant increase (doubling) in the number of apoptotic cells 48 hours after radiotherapy (absolute increase of 14.7% in MDA-MB-231 cells and 8.0% increase in CAL-120 cells compared with radiotherapy alone; Fig. 2A and B). In addition, ABT-263 pretreatment before radiotherapy (4 Gy) increased the formation of cleaved PARP and cleaved caspase 3, two protein markers of apoptosis, at 48 hours after radiotherapy compared with either radiotherapy or drug alone in both MDA-MB-231 and CAL-120 cells (Fig. 2A and B).

Alternatively, ABT-263 with radiotherapy (4 Gy) did not lead to a significant increase in apoptotic cells compared with radiotherapy alone in PTEN-null cell lines (Fig. 2C and D) or *PIK3CA*-mutant cells (Fig. 2E). In all three cell lines (MDA-MB-468, CAL-51, SUM-159), ABT-263 + RT also failed to increase cleaved PARP and cleaved caspase 3 formation, even demonstrating a slight decrease in cleaved PARP formation. Thus, combined inhibition of Bcl-2 and Bcl-xL using the nonspecific inhibitor ABT-263 led to increased apoptosis and radiosensitization in a *PIK3CA/PTEN* pathway–dependent manner. Because *PIK3CA/PTEN* wild-type cell lines express higher levels of Bcl-2 and Bcl-xL and lower levels of Mcl-1 protein compared with *PIK3CA/PTEN*-mutant TNBC cell lines (Fig 2F), we next sought to assess the contributions of individual Bcl-2 family proteins to the radiosensitization phenotype.

### Specific Inhibition of Bcl-xL, but not Bcl-2, Radiosensitizes *PIK3CA/PTEN* Wild-type TNBC

**FIGURE 3:**
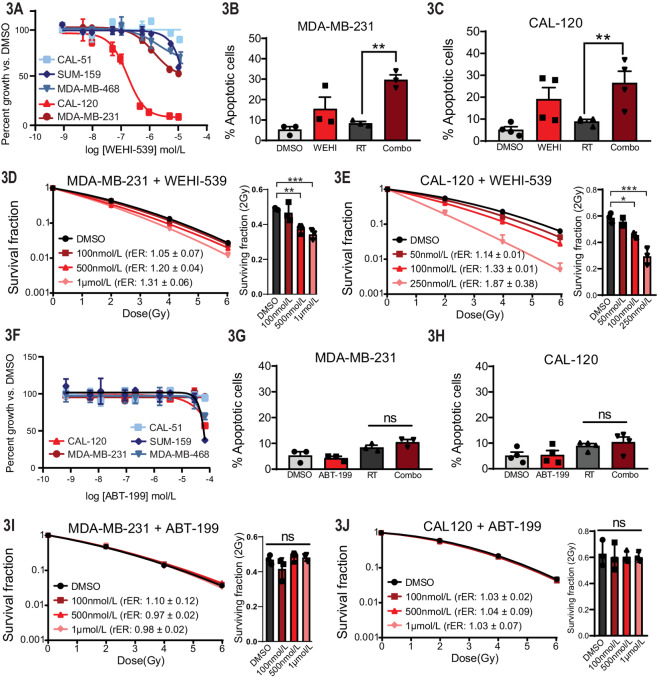
Bcl-xL, but not Bcl-2, is responsible for radiosensitivity in PIK3CA/PTEN wild-type TNBC. Cellular viability (*n* = 6 technical replicates and *n* = 3 biological replicates) was assessed in *PIK3CA/PTEN* wild-type (red) and mutant (blue) TNBC cell lines following 72-hour treatment with WEHI-539 (**A**) or ABT-199 (**F**). Annexin V/PI flow cytometry (*n* = 3–4 biological replicates) was used to quantify the number of apoptotic cells following drug and/or radiotherapy treatment (**B, C, G, H**) and clonogenic survival assays (*n* = 3 biological replicates) were used to quantify the effects of combined RT + WEHI-539 (**D** and **E**, 1 μmol/L pretreatment for MDA-MB-231 and 250 nmol/L pretreatment for CAL-120) or ABT-199 (**I** and **J**, 1 μmol/L for both cell lines) in *PIK3CA/PTEN* wild-type TNBC. *t* tests were used to compare radiotherapy and combination treated groups and a one-way ANOVA with Dunnett *post hoc* test was used to compare SF-2Gy values within each cell line (ns, not significant; *, *P* < 0.05; **, *P* < 0.01; ***, *P* < 0.001).

To determine the effect of inhibiting individual Bcl-2 protein family members on *PIK3CA/PTEN* wild-type TNBC cell lines, we used specific pharmacologic inhibitors targeted against Bcl-2 family members: WEHI-539, a Bcl-xL–specific inhibitor (and the orally bioavailable analog, A-1331852; ref. 44); ABT-199 (venetoclax), a Bcl-2–specific inhibitor (45); and the Mcl-1 inhibitor S63845 (32). To assess Bcl-xL inhibitor-mediated effects on cell viability, we treated TNBC cell lines with 1 nmol/L-10 μmol/L WEHI-539 for 72 hours (Fig. 3A; Supplementary Table S2). Single-agent effects of WEHI-539 on cell growth were dependent on *PIK3CA/PTEN* mutational status *in vitro*, with *PIK3CA/PTEN*-mutant cell lines demonstrating resistance to Bcl-xL inhibition, which mirrored the lack of *in vitro* effects that were observed with the pan inhibitor ABT-263. Combined WEHI-539 and radiotherapy led to an increase in the number of apoptotic cells (Fig. 3B and C) and cleaved PARP formation (Supplementary Fig. S1A) in both MDA-MB-231 and CAL-120 cells compared with 4 Gy radiotherapy alone (22.3% and 17.6% increases in the fraction of apoptotic cells, respectively).

Using clonogenic survival assays, we determined that the Bcl-xL inhibitor WEHI-539 led to clinically relevant levels of radiosensitization (rER > 1.2) in *PIK3CA/PTEN* intact TNBC (MDA-MB-231 and CAL-120; Fig. 3D and E) that were comparable with the effects of combined ABT-263 pretreatment with radiotherapy (MDA-MB-231 rER 1 μmol/L: 1.31 ± 0.06; CAL-120 rER 1 μmol/L: 1.87 ± 0.38). Consequently, combined WEHI-539 treatment and radiotherapy led to a concentration-dependent decrease in the SF-2Gy in both cell lines. To further confirm that Bcl-xL inhibition was responsible for radiosensitization of *PIK3CA/PTEN* wild-type TNBC cell lines, we repeated the clonogenic survival assays with A-1331852, an analog of WEHI-539 developed for *in vivo* use (44, 46). As with WEHI-539, treatment with A-1331852 radiosensitized MDA-MB-231 and CAL-120 cells (MDA-MB-231 rER 1 μmol/L: 1.32 ± 0.07; CAL-120 rER 1 μmol/L: 2.00 ± 0.47) and significantly increased cleaved PARP formation in combination treated groups (Supplementary Fig. S1B–S1D). In addition, to quantify the potential normal tissue toxicity induced by pan Bcl-2 family inhibition or Bcl-xL inhibition, we performed clonogenic survival assays in immortalized normal mammary epithelial cells (MCF-10A) and calculated the resulting rERs (Supplementary Fig. S1E). Neither ABT-263, WEHI-539, nor A-1331852 induced any radiosensitization at a concentration of 500 nmol/L, suggesting that Bcl-xL inhibition + RT is not likely to induce significant toxicity or apoptosis in normal breast tissue.

Having established that Bcl-xL inhibition was sufficient to confer radiosensitivity to *PIK3CA/PTEN* wild-type TNBC models, we next sought to determine whether Bcl-2 also contributed to this radiosensitization. Unlike ABT-263 and WEHI-539, which suppressed growth of *PIK3CA/PTEN* wild-type TNBC cell lines, the Bcl-2–specific inhibitor ABT-199 had no effect on TNBC cell line viability (regardless of *PIK3CA/PTEN* mutational status) at doses less than 5 μmol/L (Fig. 3F; Supplementary Table S2). Not surprisingly, ABT-199 did not lead to increased apoptosis in MDA-MB-231 or CAL-120 cells (Fig. 3G and H) nor did it lead to radiosensitization at concentrations up to 1 μmol/L (MDA-MB-231 rER: 0.98 ± 0.02; CAL-120 rER: 1.03 ± 0.07; Fig. 3I and J). Finally, the combination of ABT-199 and radiotherapy (4 Gy) did not increase cleaved PARP levels compared with either radiotherapy or drug alone (Supplementary Fig. S1F). As expected, ABT-199, WEHI-539, and A-1331852 all failed to induce significant radiosensitization in *PIK3CA/PTEN*-mutant TNBC cell lines (MDA-MB-468, SUM-159, CAL-51; Supplementary Fig. S2A–S2C).

Although ABT-263 is a less potent inhibitor of Mcl-1 compared with Bcl-2 or Bcl-xL, Mcl-1 has been suggested as a potential therapeutic target in TNBC in multiple studies (32, 47); therefore, we sought to assess the effects of the Mcl-1–specific inhibitor S63845 (32) on radiosensitization in our *in vitro* models of TNBC. Pretreatment with S63845 did not significantly inhibit proliferation of TNBC cell lines (Supplementary Fig. S3A; Supplementary Table S2), though modest effects were seen in *PIK3CA/PTEN*-mutant cell lines at high concentrations, consistent with prior literature (32). S63845 failed to induce radiosensitization in *PIK3CA/PTEN* wild-type CAL-120 cells (Supplementary Fig. S3B,; rER: 1.06 ± 0.08) or *PIK3CA*-mutant SUM-159 cells (Supplementary Fig. S3C; rER: 0.95 ± 0.10), or *PIK3CA/PTEN*-mutant CAL-51 cells (Supplementary Fig. S3D; rER: 1.07 ± 0.10), suggesting that Mcl-1 inhibition alone did not potentiate apoptosis in either *PIK3CA/PTEN* wild-type or mutant TNBC cells.

### Bcl-xL Inhibition Radiosensitizes *PIK3CA/PTEN* Wild-type TNBC Xenograft Tumors

**FIGURE 4:**
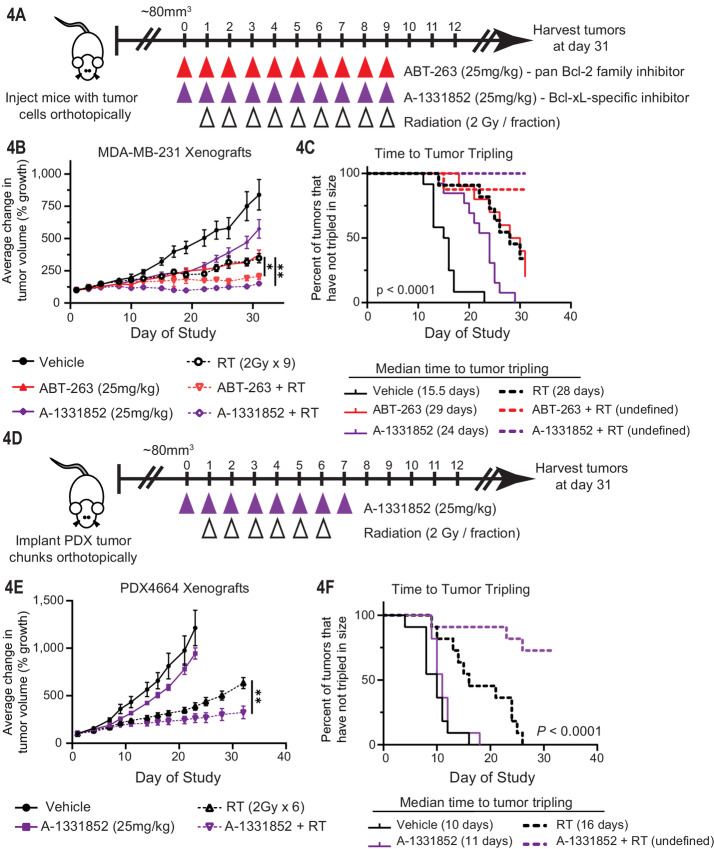
Pan Bcl-2 family inhibition or specific inhibition of Bcl-xL radiosensitizes *PIK3CA/PTEN* wild-type TNBC xenografts. MDA-MB-231 xenograft tumors (**A**) were treated with 25 mg/kg ABT-263 or 25 mg/kg A-1331852 ± concurrent radiotherapy with 13–16 tumors per group. Tumor volume was measured every 2–3 days (**B**) and used to calculate the median time to tumor tripling (**C**). A PDX model of TNBC was also used to assess effects of A-1331852 + RT (**D, E,** and **F**) with 11 tumors per group. Tumor volume was compared using a one-way ANOVA at the study endpoint and Kaplan–Meier curves were compared using the log-rank (Mantel–Cox) test. (*, *P* < 0.05; **, *P* < 0.01; ****, *P* < 0.0001).

To examine the effects of Bcl-xL inhibitor–mediated radiosensitization *in vivo*, we generated MDA-MB-231 xenograft tumors by injecting cells into the mammary fat pads of female SCID CB-17 mice (13–16 tumors per group). Following the formation of established tumors (∼80 mm^3^) and randomization, mice were assigned to receive either 25 mg/kg of ABT-263 (pan Bcl-2 family inhibitor), 25 mg/kg of A-1331852 (Bcl-xL inhibitor), nine fractions of 2 Gy radiotherapy, or a combination of either ABT-263 or A-1331852 with radiotherapy. In combination-treated mice, radiotherapy treatment started 24 hours after the first treatment with drug, and drug was given for a total of 10 days (Fig. 4A). All treatment was discontinued after the ninth fraction of radiotherapy. Overall, pan Bcl-2 family inhibition with ABT-263 or specific Bcl-xL inhibition with A-1331852 and radiotherapy significantly decreased tumor growth compared with drug or radiotherapy alone (Fig. 4B) and significantly extended time to tumor tripling (Fig. 4C).

In addition to a cell line–derived xenograft model, we tested the effects of Bcl-xL inhibition in a PDX model of wild-type *PIK3CA/PTEN* TNBC using a similar treatment paradigm (Fig. 4D, 11 tumors per group). In this PDX model, combination treatment with A-1331852 and radiotherapy significantly decreased tumor growth (Fig. 4E) and time to tumor tripling (Fig. 4F) compared with either single treatment arm alone. Although the effects of ABT-263 were only additive with radiotherapy in cell line xenografts (FTV ratio < 1; Supplementary Table S3), A-1331852 was synergistic with radiotherapy in both MDA-MB-231 and PDX4664 xenografts (FTV ratio > 1; Supplementary Tables S4 and S5). Finally, in both the cell line and PDX studies, combination therapy with ABT-263 or A-1331852 and radiotherapy did not lead to significant toxicity or weight loss (Supplementary Fig. S4) in mice compared with other treatment groups, suggesting that the combination therapy is relatively well tolerated.

### *PTEN* Knockout Leads to Increased Mcl-1 Expression and Radioresistance

**FIGURE 5:**
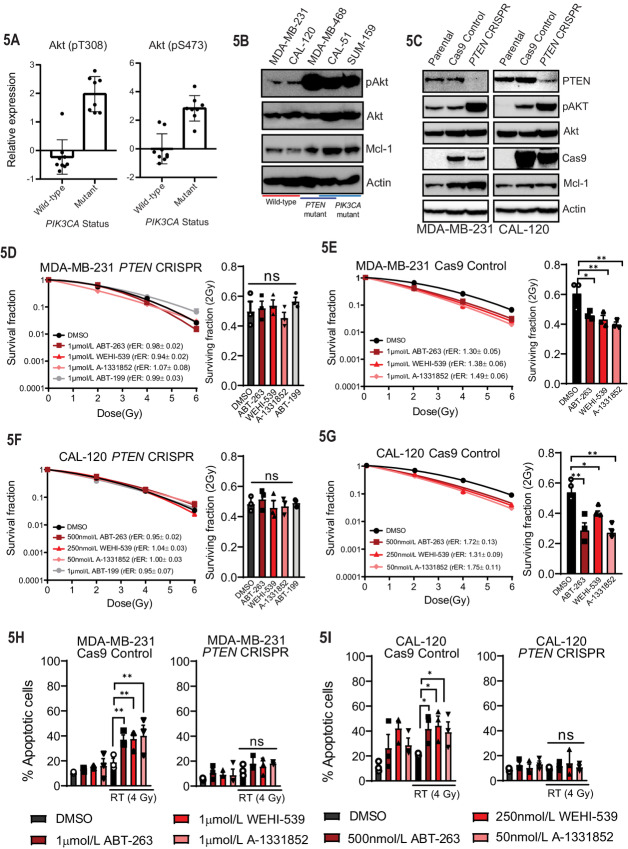
*PTEN* loss leads to increased Akt/Mcl-1 expression and abolishes radiosensitization in TNBC cell lines. CCLE data were used to plot pAkt expression based on *PIK3CA* mutation status in TNBC cell lines (**A**). Western blots (*n* = 3 biological replicates) were used to assess pAkt, Akt, and Mcl-1 expression at baseline (**B**) and following Cas9-mediated knockout of *PTEN* in MDA-MB-231 and CAL-120 cells (**C**). Clonogenic survival assays were performed in Cas9 control and *PTEN* CRISPR knockout cells to assess radiosensitivity with ABT-263, WEHI-539, A-1331852, and ABT-199 (**D, E, F, and G,***n* = 3 biological replicates). Annexin V/PI-based flow cytometry (*n* = 3 biological replicates) was used to quantify apoptosis 48 hours after combination treatment in Cas9 control and *PTEN* knockout TNBC cell lines (**H** and **I**). (ns, not significant; *, *P* < 0.05; **, *P* < 0.01).

To further understand how PI3K/PTEN signaling contributes to Bcl-xL inhibitor–mediated radiosensitization of TNBC, we next sought to understand the cellular changes induced by PI3K pathway mutations in these models (Fig. 5). Using the Cancer Cell Line Encyclopedia, we analyzed expression of phosphorylated Akt (pAKT T308 and S473), a signaling mediator downstream of activated PI3K, across breast cancer cell lines with either wild-type PI3K signaling or activating mutations in the PI3K pathway. As expected, hyperactivation of PI3K signaling resulted in higher expression of pAkt, which we confirmed in our cell line models (Fig. 5A–B). To understand the effects of *PTEN* loss in our models, we used CRISPR-Cas9 to generate isogenic models of MDA-MB-231 and CAL-120 with *PTEN* knockout (Fig. 5C).

In these models, knockout of the *PTEN* tumor suppressor gene led to a baseline increase in pAkt (Ser473) and Mcl-1 expression in *PTEN* knockout cell lines compared with parental or Cas9 control cells. We assessed the effects of PTEN loss on Bcl-xL inhibitor–mediated radiosensitivity by repeating the clonogenic survival assays in *PTEN* knockout cells and Cas9-expressing CRISPR control TNBC cell lines. In MDA-MB-231 *PTEN* knockout cells, drug pretreatment failed to sensitize cells to radiotherapy when 1 μmol/L ABT-263 (rER: 0.98 ± 0.02), 1 μmol/L WEHI-539 (rER: 0.94 ± 0.02), or 1 μmol/L A-1331852 (rER: 1.07 ± 0.08) was given 1 hour prior to radiotherapy (Fig 5D). Similar results were achieved with CAL-120 cells (rER: 0.95 ± 0.02 with 500 nmol/L ABT-263, rER: 1.04 ± 0.03 with 250 nmol/L WEHI-539, and rER: 1.00 ± 0.03 with 50 nmol/L A-1331852; Fig. 5F). The Bcl-2–specific inhibitor ABT-199, which did not lead to radiosensitization in *PTEN* wild-type parental cell lines, remained unable to induce radiosensitization in the *PTEN* knockout models (rER: 0.95–0.99).

Consistent with our previous results, isogenic control cell lines expressing the Cas9 protein with a control (*AAVS1)* gRNA were radiosensitized by pan Bcl-2 family inhibition (ABT-263) and Bcl-xL–specific inhibition at magnitudes similar to the parental (non-CRISPR) cell lines [ABT-263 (rER: 1.30–1.72), WEHI-539 (rER: 1.31–1.38) or 1 μmol/L A-1331852 (rER: 1.49–1.75)] (Fig. 5E–G), suggesting that the observed effect on radiosensitivity is dependent on loss of *PTEN*. Mechanistically, *PTEN* knockout abolished Bcl-xL inhibitor–mediated induction of apoptosis following radiotherapy in MDA-MB-231 and CAL-120 cells (Fig. 5H and I), but Cas9 control cells remained sensitive to the proapoptotic effects of pan Bcl-2 family inhibition (ABT-263) and Bcl-xL–specific inhibition (WEHI-539 and A-1331852).

**FIGURE 6:**
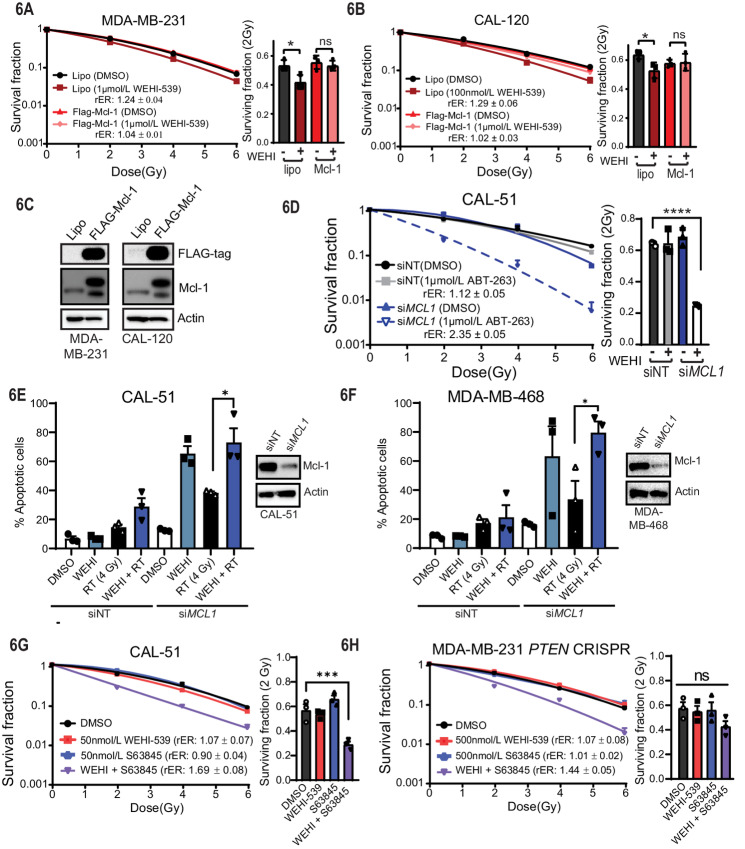
Mcl-1 expression leads to radioresistance in TNBC cell lines. Radiosensitization of cells with transient Mcl-1 overexpression (*n* = 3 biological replicates) was assessed in MDA-MB-231 (**A**) and CAL-120 cells (**B**) 24 hours after transfection of FLAG-Mcl-1. Western blots were used to verify Mcl-1 protein expression 24 hours after transfection of siRNA or FLAG-Mcl-1 (**C**) and radiosensitization was assessed when si*MCL1* was used to transiently knockdown *MCL1* expression in CAL-51 cells (**D**, *n* = 3 biological replicates). Cells were treated with 1 μmol/L ABT-263 or 1 μmol/L WEHI-539 24 hours after transfection (si*MCL1*) and harvested 48 hours after radiotherapy for Western blots or Annexin V/PI-based flow cytometry (**E** and **F**) to assess apoptosis (*n* = 3 biological replicates). Clonogenic assays (*n* = 3 biological replicates) were used to quantify radiosensitization with combined WEHI-539 + S63845 treatment (**G** and **H**). (ns, not significant; *, *P* < 0.05; ***, *P* < 0.001; ****, *P* < 0.0001).

Increased Akt signaling has been shown to lead to increased translation and expression of Mcl-1 (20). In our models, we hypothesized that higher expression of Akt/Mcl-1 in *PIK3CA/PTEN*-mutant cell lines conferred radioresistance, and that we could induce radioresistance in *PIK3CA/PTEN* wild-type cells with transient Mcl-1 overexpression. In these models, transient Mcl-1 overexpression prevents WEHI-539–mediated radiosensitization of MDA-MB-231 (rER: 1.04 ± 0.01) and CAL-120 cells (rER: 1.02 ± 0.03; Fig. 6A–C). Overexpression of Mcl-1 in MDA-MB-231 cells also led to a reduction in PARP1 cleavage following radiotherapy + WEHI-539 (Supplementary Fig. S5A), suggesting that Mcl-1 expression induces radioresistance.

Conversely, *MCL1* knockdown led to increased cleaved PARP formation (Supplementary Fig. S5B), increased radiosensitization (Fig. 6D; rER: 2.35 ± 0.05) and increased apoptosis (Fig. 6E) with combined Bcl-xL inhibitor therapy and radiotherapy in *PIK3CA/PTEN*-mutant CAL-51 cells. Similar results were found with *MCL1* knockdown in PTEN null MDA-MB-468 cells (Fig. 6F), suggesting that Mcl-1 is a modulator of radioresistance in TNBC. This was further confirmed with pharmacologic inhibitors of Bcl-xL and Mcl-1 in *PIK3CA/PTEN*-mutant TNBC. Although low concentrations of WEHI-539 and S63845 are insufficient to produce radiosensitization in isolation, the combination of Bcl-xL inhibition and Mcl-1 inhibition leads to radiosensitization in CAL-51 cells (rER: 1.69 ± 0.08; Fig. 6G) and in MDA-MB-231 *PTEN* CRISPR cells at radiotherapy doses higher than 2 Gy (rER: 1.44 ± 0.05; Fig. 6H).

We also validated these changes in radiosensitivity through modulation of the upstream modulator, Akt, expression, in *PIK3CA/PTEN*-mutant cell lines (Supplementary Fig. S6). Following *AKT1* knockdown by siRNA, Bcl-xL inhibition radiosensitized *PIK3CA*-mutant CAL-51 cells (WEHI-539 1 μmol/L: rER: 1.46 ± 0.06; Supplementary Fig. S6A and S6D). *AKT1* knockdown did not induce global sensitivity to Bcl-2 family inhibitors, as the Bcl-2–specific inhibitor ABT-199 remained unable to radiosensitize CAL-51 cells (rER: 0.95 ± 0.07) despite *AKT1* knockdown (Supplementary Fig. S6B). Finally, although we previously demonstrated that *PTEN* knockout abrogates Bcl-xL inhibitor–mediated radiosensitzation in MDA-MB-231 cells, rescue experiments with the addition of *AKT1*-targeting siRNA partially restores radiosensitization (Supplementary Fig. S6C and S6D; rER: 1.37 ± 0.03). Although these changes were more pronounced at higher radiotherapy doses (4–6 Gy compared with 2 Gy), these results further support the hypothesis that manipulation of the Akt/Mcl-1 signaling axis is sufficient to modulate Bcl-xL inhibitor–mediated radiosensitization in TNBC.

### Mcl-1 Signaling Induces Resistance to Bcl-xL Inhibitor–mediated Radiosensitization in TNBC Through Increased Activation of Bak

**FIGURE 7:**
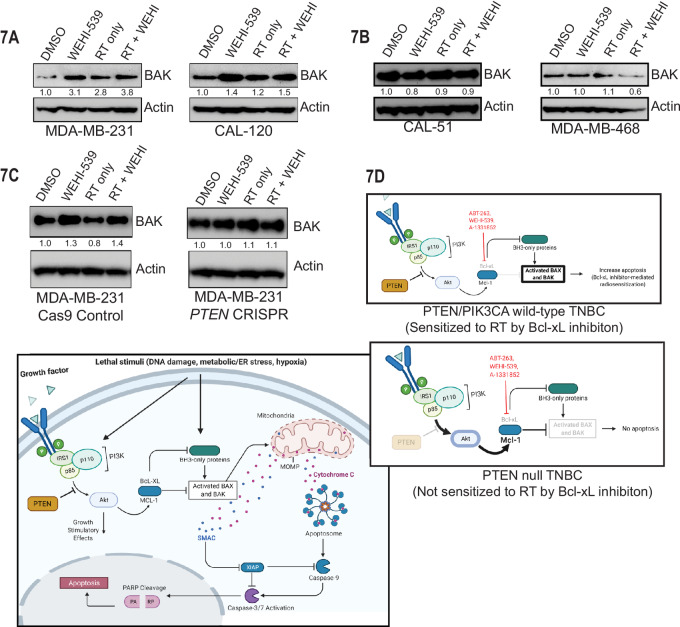
Bcl-xL inhibitor–mediated radiosensitivity results in increased Bak expression in TNBC. Expression of Bak was assessed 24 hours after radiotherapy (1 hour drug pretreatment before radiotherapy, *n* = 3 biological replicates) in *PIK3CA/PTEN* wild-type (MDA-MB-231, CAL-120, MDA-MB-231 Cas9 control) and mutant (CAL-51, MDA-MB-468, MDA-MB-231 *PTEN* CRISPR) cell lines (**A–C**). In *PIK3CA/PTEN* wild-type cell lines, Bcl-xL inhibition leads to radiosensitization through suppression of Bcl-xL activity and increased Bak expression. In *PIK3CA/PTEN*-mutant type cell lines, overactivation of Akt leads to increased Mcl-1 activation and minimal induction of Bak, preventing Bcl-xL inhibitor–mediated radiosensitization (**D**).

To further elucidate the connection between increased Mcl-1 and increased apoptosis following treatment with Bcl-xL inhibition + RT, we assessed the expression of proapoptotic proteins such as Bcl-2 homologous antagonist killer (Bak) in TNBC cell lines. Bak protein was significantly elevated following treatment with WEHI-539 or WEHI-539 + RT in CAL-120 and MDA-MB-231 cells (Fig. 7A). Induction of BAK did not occur in radioresistant *PTEN/PIK3CA*-mutant CAL-51 or MDA-MB-468 cells (Fig. 7B). When comparing isogenic models of *PTEN* loss in MDA-MB-231 cells, induction of Bak expression occurred in Cas9 control cells after treatment with WEHI-539 ± RT but failed to occur in MDA-MB-231 *PTEN* CRISPR cells (Fig. 7C).

On the other hand, expression of the apoptotic mediator BAX does not increase as a result of Bcl-xL inhibition + RT (Supplementary Fig. S7) or combination treatment with radiotherapy or any of the other Bcl-2 family inhibitors. Although expression of Bcl-xL does not significantly change as a result of combination treatment regardless of *PIK3CA/PTEN* status, Mcl-1 expression is induced in response to treatment with S63845, particularly in *PIK3CA/PTEN* wild-type cell lines, whereas Bcl-2 induction occurs primarily in *PIK3CA/PTEN*-mutant TNBC (Supplementary Fig. S7). Taken together, our results suggest that *PIK3CA/PTEN* wild-type TNBC cell lines can be radiosensitized through inhibition of Bcl-xL, but *PIK3CA/PTEN*-mutant cell lines that overexpress Akt/Mcl-1 cannot properly induce Bak expression which may be responsible, at least in part, for apoptotic cell death in response to radiotherapy (Fig. 7D).

## Discussion

In this study, we describe the identification of a targeted approach that may be useful in increasing the efficacy of radiotherapy in aggressive, radioresistant, TNBCs. First, we demonstrated that treating *PIK3CA/PTEN* wild-type TNBC with either a pan Bcl-2 family inhibitor (ABT-263; Fig. 1) or specific inhibitors of Bcl-xL (WEHI-539, A-1331852)—but not specific inhibitors of Bcl-2 or Mcl-1—resulted in radiosensitization by potentiating radiotherapy-induced apoptotic cell death (Figs. 2 and 3). Pan-Bcl-2 family inhibition or Bcl-xL–specific inhibition combined with radiotherapy *in vivo* led to significantly reduced tumor sizes and delayed tumor growth in cell line and PDX TNBC models (Fig. 4). Finally, we show that in TNBC cell lines with activating PI3K pathway mutations (either *PIK3CA* mutations or *PTEN* loss), radioresistance occurs through increased basal levels of Akt and Mcl-1 and cellular apoptosis that occurs in *PIK3CA/PTEN* wild-type TNBC (Figs. 5 and 6). Together, our results provide preclinical data in support of Bcl-xL inhibition as a potential clinical strategy for radiosensitization of *PIK3CA/PTEN* wild-type breast cancers with low expression of Mcl-1.

Currently, the primary therapeutic modalities for TNBC are surgery, radiotherapy, and cytotoxic chemotherapy—and in some cases the anti-PD-1 antibody pembrolizumab. Although we focused on the use of Bcl-xL inhibition in combination with radiotherapy, others have demonstrated parallel interactions *in vitro* between BH3 mimetics and chemotherapeutic agents. The antineoplastic agent docetaxel is synergistic with the Mcl-1 inhibitor S63845 in TNBC and HER2-amplified breast cancers (32), the first-generation pan Bcl-2 family inhibitor ABT-737 in ER^+^ breast cancer (33), and the Bcl-xL–specific inhibitor A-1331852 in a wide range of solid tumor types (46). In addition, targeting Bcl-2 family proteins with BH3 mimetics such as ABT-737 and ABT-263 also sensitizes TNBC cells to other taxols including paclitaxel (48, 49). In combination with anthracycline chemotherapies, nuclear pAkt has been shown to predict the efficacy of PI3K and doxorubicin in breast and ovarian cancers. Furthermore, ABT-263 leads to selective cell death in *TP53* wild-type breast cancers after the induction of doxorubicin-induced senescence (50) and activating *PIK3CA* mutations confer resistance to chemotherapies in TNBC through increased Akt/mTOR signaling and a subsequent reduction in apoptosis (51). A major issue with targeting important signaling pathways for anticancer treatment is the reliance of normal cells on the activity of those pathways and the subsequent normal tissue toxicities. Our data suggest that single-agent Bcl-xL has limited single-agent effects, but the combination with radiation makes these compounds particularly effective in *PIK3CA/PTEN* wild-type TNBC. As radiotherapy is delivered with accuracy to the millimeter, normal tissue toxicity can be effectively limited, as dose to heart, lung, spinal cord, and other organs can be essentially eliminated. Thus, although radiotherapy, like systemic chemotherapy, can induce cytotoxic effects in tumor cells, the conformal nature of radiotherapy for the treatment of breast cancer can effectively reduce the risk to healthy organs and tissues; this suggests that our proposed combination therapy may be significantly less toxic than combination therapies using systemic chemotherapies.

Our data support a growing body of literature that suggests that the role of each Bcl-2 family protein is determined in a context-dependent manner, leading to differential regulation of Bcl-xL, Mcl-1, and Bcl-2 expression across different cancer types. Our models support the current hypothesis that increased Akt signaling drives Mcl-1 expression (21, 52–54) and that inhibition of PI3K/Akt signaling results in downregulation or degradation of Mcl-1 (22, 23, 55, 56). Interestingly, in our models, the *PIK3CA/PTEN*-mutant TNBC cell lines also have the lowest expression of Mcl-1, which could contribute to the Bcl-xL inhibitor–mediated radiosensitization phenotype seen in this study. Pharmacologic Mcl-1 inhibition alone with S63845 does not radiosensitize TNBC cell lines, which is likely due, at least in part, to an induction of Mcl-1 activity that occurs after short-term treatment with S63845.

It has also been shown that dual targeting of Bcl-xL and PI3K in *PIK3CA*-mutant breast cancer models blocks tumor growth *in vivo* through modulation of mTOR-mediated Mcl-1 translation (20); this is consistent with our observation that blocking PI3K signaling (through genetic knockdown of *AKT1* or *MCL1*) renders *PIK3CA/PTEN*-mutant TNBC models sensitive to Bcl-xL inhibitor–mediated radiosensitivity. In this study, we did not directly quantify the total amount of Bcl-2 family proteins in the cell, nor did we calculate the relative proportions of each of the Bcl-2 family proteins. Thus, further studies (including metabolic amino acid labeling or immunoprecipitation) would allow us to quantify and compare absolute protein levels for each of the Bcl-2 family proteins in our TNBC cell line models.

Despite many studies exploring Bcl-2 family proteins in breast cancer, most of the current literature has focused on the role of the antiapoptotic protein Mcl-1 (32, 47) in examining the efficacy of combination therapies using BH3 mimetics with other targeted agents such as NVP-BEZ235, everolimus (RAD001), and other pharmacologic inhibitors that target mTOR or PI3K signaling (23, 32, 43, 57). Our studies extend this incomplete examination and describe a role for both Bcl-xL and Mcl-1 in mediating radiosensitivity in TNBC in a PI3K pathway–dependent manner. In addition, the synthetic vulnerability of *PTEN* loss and pharmacologic Mcl-1 inhibition has been explored in the context of *PTEN*-deficient models of glioblastoma (54), but our study is the first to demonstrate that both *PIK3CA* mutations and *PTEN* loss in breast cancer cells can induce resistance to Bcl-xL inhibitor–mediated radiosensitivity. As noted, patients with *PI3KCA* and *PTEN* wild-type TNBC have increased rates of locoregional and overall recurrence after radiation (24) We validated this in a separate dataset (40), where our analysis confirmed that outcomes are worse for patients with *PIK3CA/PTEN* wild-type TNBC despite receiving radiotherapy treatment as part of the standard of care (Supplementary Fig. S8). Thus, combinations that selectively target this pathway in this patient population might be of clinical benefit. Indeed, clinical practice is increasingly moving toward upfront sequencing of tumors using commercial platforms that report *PTEN* and *PIK3CA* mutational status (58, 59). Subject to clinical validation, this information may be useful in deciding when to utilize a radiosensitizer like a Bcl-xL inhibitor in women with TNBC at high risk or locoregional recurrence based on clinicopathologic features (large tumor, lymph node involvement, high-grade disease, etc.).

Although we focused on aggressive, radioresistant models of TNBC in this study, future studies in our laboratory are underway to determine the effects of Bcl-xL inhibition in other breast cancer subtypes. These ongoing studies will allow us to elucidate the potential for context-dependent differences in Bcl-2 family inhibitor–mediated radiosensitization across a more heterogenous population of breast cancer models and would build on recent literature demonstrating differences in sensitivity to Bcl-2 family inhibitors across different breast cancer subtypes (20, 32, 42, 43). In addition, expanding these studies using additional breast cancer models will allow us to explore the distinct functions of other Akt isoforms (60) in the context of Akt-mediated expression of Bak and Bcl-xL–mediated radiosensitization.

Taken together, our results suggest that Bcl-xL inhibition is a viable therapeutic strategy to increase the efficacy of radiotherapy when given as part of the standard of care for patients with TNBC in the absence of PI3K pathway–activating mutations. ABT-199 (venetoclax) is FDA approved for the treatment of some hematologic malignancies, but the use of BH3 mimetics targeting Bcl-xL—such as A-1331852—would need to undergo further safety and toxicity studies in combination with radiotherapy to identify and mitigate any potential overlapping toxicities. Finally, there is a growing pipeline of novel BH3 mimetics (APG-2575, BM-1197, LOXO-338, and AZD0466) among others such as Bcl-xL PROTAC degraders (PZ703b). These compounds are under preclinical and early clinical investigation (61) and, if given concurrently with ionizing radiation, have the potential to influence radiation sensitivity in a wide variety of tumor types. Although clearly awaiting further clinical evaluation and validation, in an era of increased somatic mutation testing, these data suggest that using *PIK3CA/PTEN* mutational status might be a possible biomarker of efficacy of Bcl-xL inhibition with radiation, especially in the context of women with aggressive clinical features when the risk or locoregional recurrence is considered to be unacceptably high (multiple node-positive disease, large primary tumor, high-grade disease, etc.).

## Supplementary Material

Supplementary Data Supplemental Figures (8) and Tables
