# Supplementary material for: Bcl-xL Inhibition Radiosensitizes PIK3CA/PTEN Wild-type Triple-negative Breast Cancers with Low Mcl-1 Expression
Source: Cancer Res Commun. 2022 Jul 20;2(7):679–93. doi: 10.1158/2767-9764.CRC-22-0024 (PMC9648413; doi:10.1158/2767-9764.CRC-22-0024)
Supplement: Supplementary Data — Supplemental Figures (8) and Tables [file crc-22-0024-s01.pdf]

Supplemental Figure 1

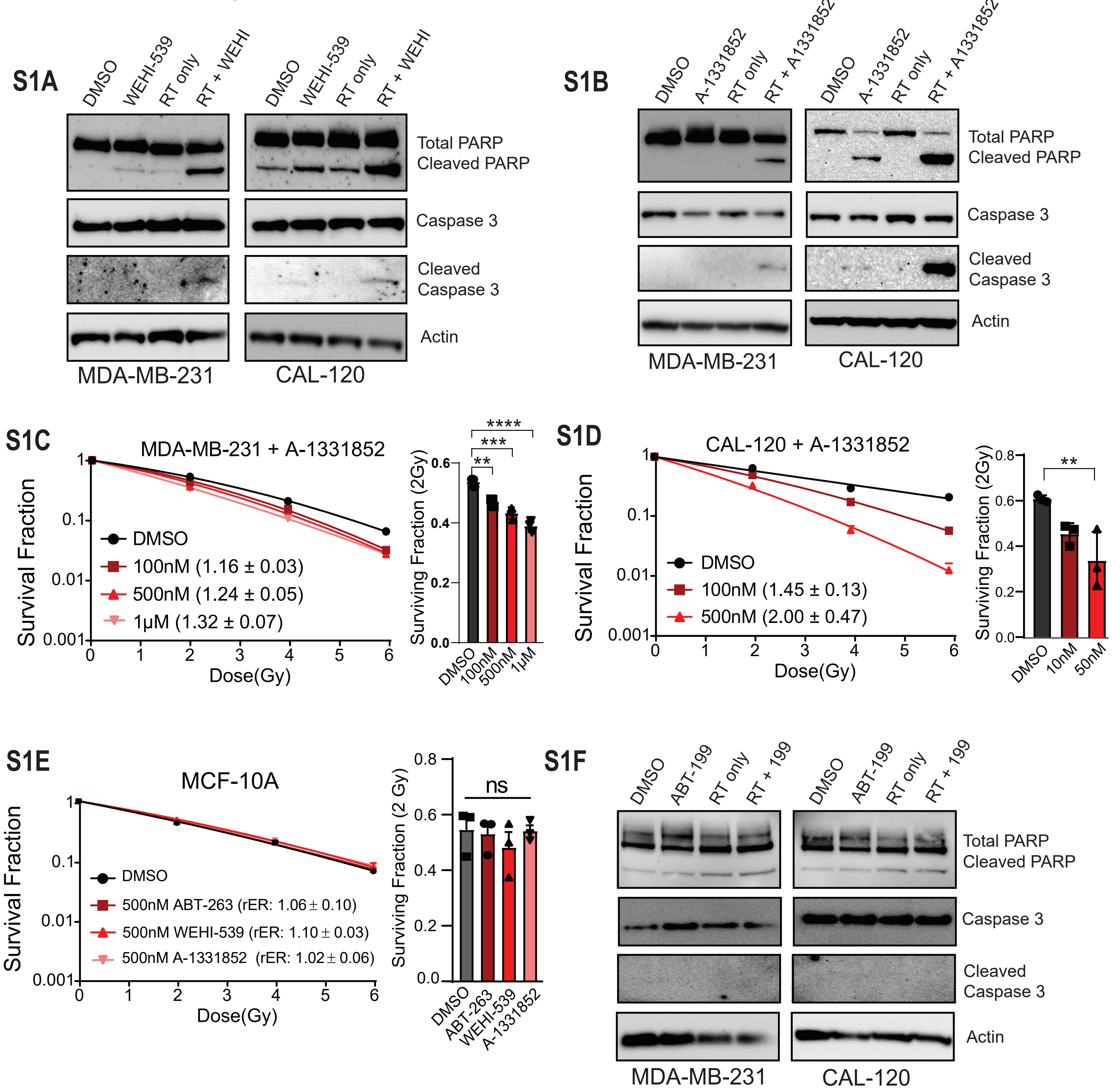

Supplemental Figure S1: **<u>Bcl-xL inhibition induces apoptosis in *PIK3CA/PTEN* wild-type TNBC</u>.** Apoptosis was assessed 48 hours after RT in MDA-MB-231 and CAL-120 cells pretreated for one-hour with WEHI-539 (1µM for MDA-MB-231 and 500nM for CAL-120), A-1331852 (1µM for MDA-MB-231 and 500nM for CAL-120), or 1µM ABT-199 prior to RT (**A,B,F**, n=3 biological replicates). Clonogenics (n=3 biological replicates) were used to assess radiosensitivity in MDA-MB-231 (**C**), CAL-120 (**D**), and MCF-10A (**E**) cells and to assess the surviving fraction of cells at 2 Gy. (ns = not significant, ** = $p < 0.01$, *** = $p < 0.001$, **** $p < 0.0001$).

Supplemental Figure 2

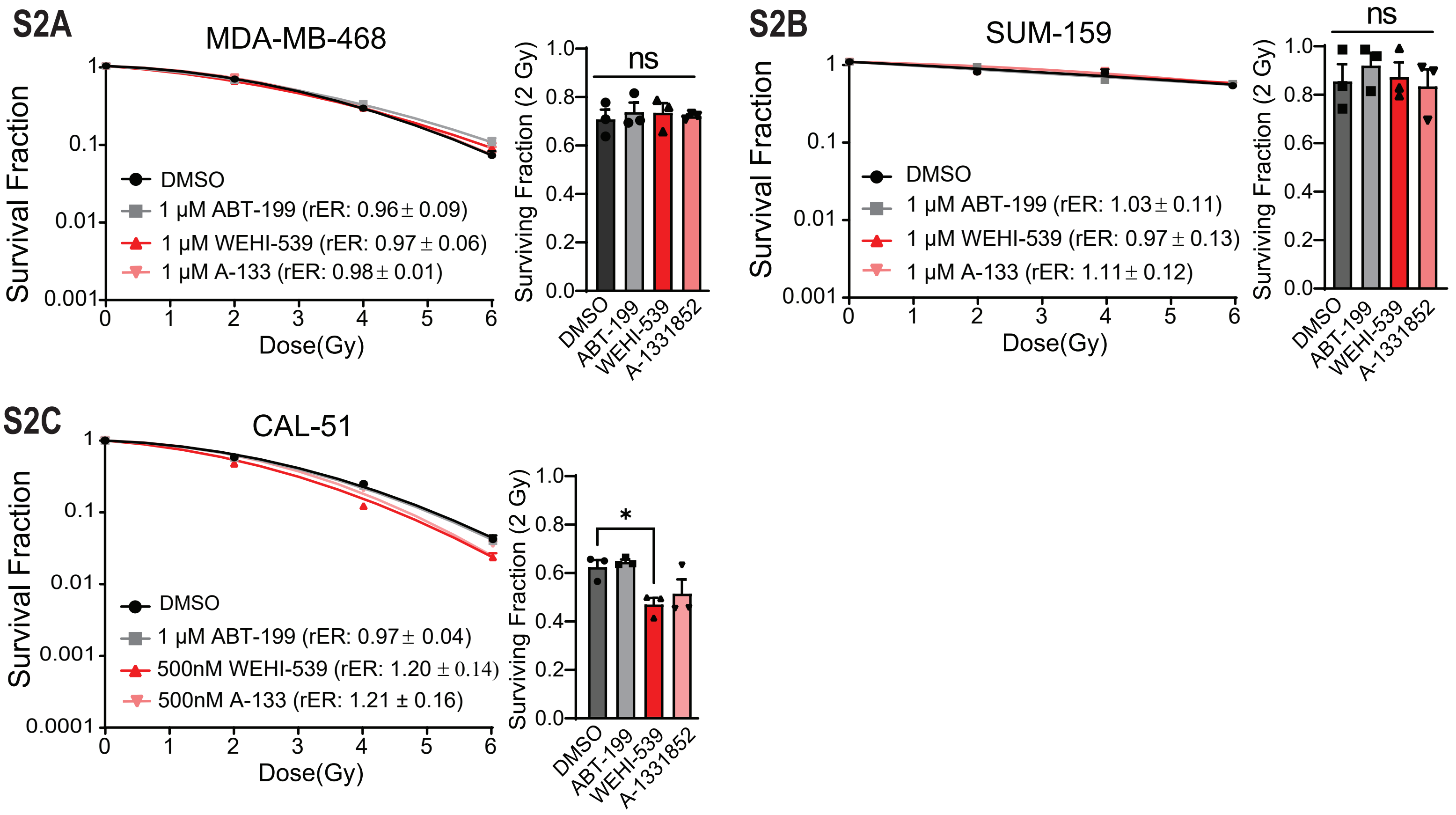

Supplemental Figure S2: <u>Bcl-xL and Bcl-2 inhibition does not radiosensitize *PIK3CA/PTEN* mutant TNBC</u>. Clonogenic survival assays (n=3 biological replicates, one hour pretreatment prior to RT) were used to quantify radiosensitization in MDA-MB-468 (**A**), SUM-159 (**B**), and CAL-51 (**C**) cells after treatment with ABT-199, WEHI-539, or A-1331852. (ns = not significant, * = $p < 0.05$)

Supplemental Figure 3

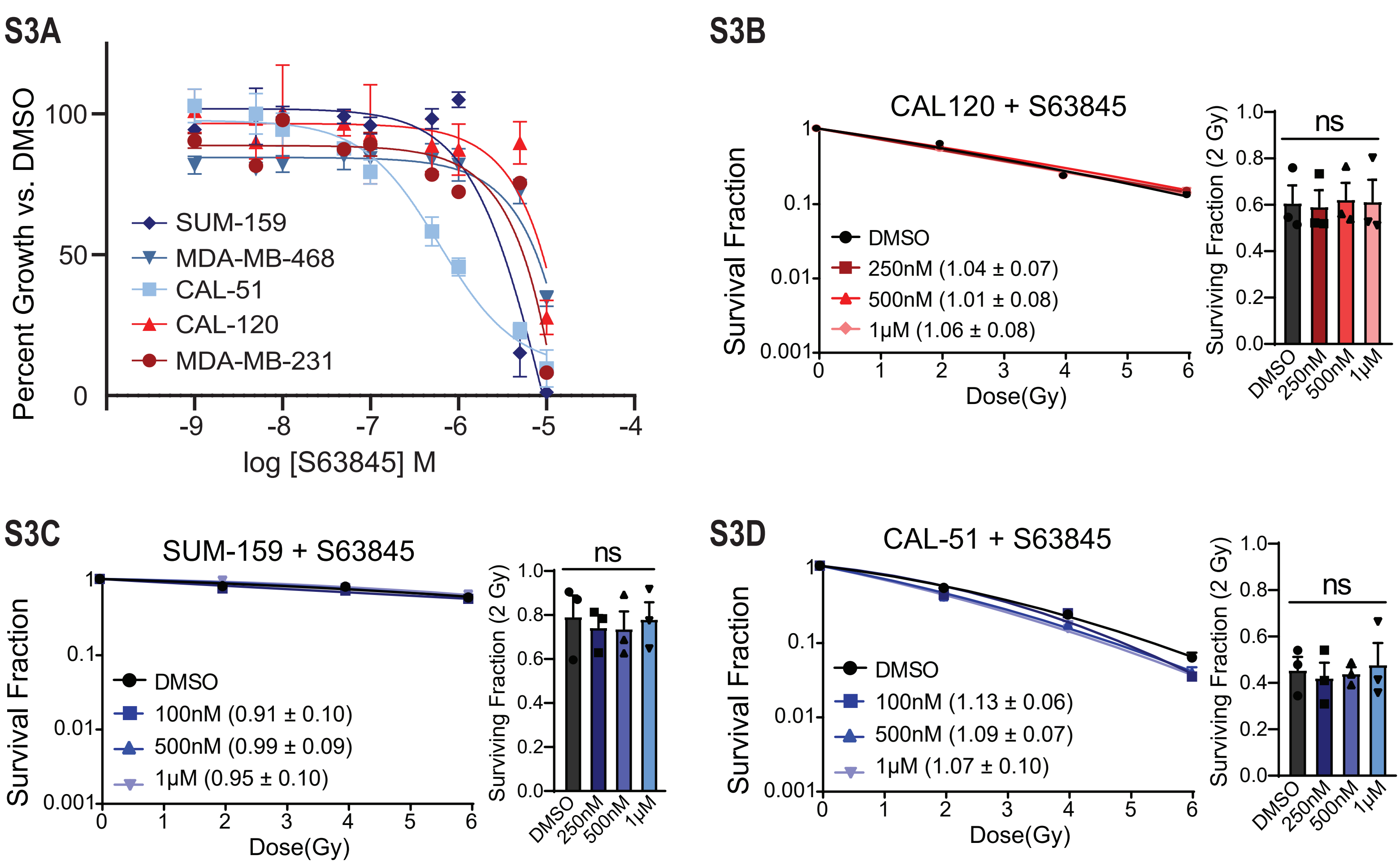

Supplemental Figure S3: <u>Mcl-1 inhibition does not radiosensitize TNBC cell lines regardless of *PIK3CA/PTEN* status.</u> Viability of TNBC cells was assessed 72 hours after treatment with the Mcl-1 inhibitor S63845 (**A**) (n=3 biological replicates). Clonogenic survival assays were performed in *PIK3CA/PTEN* wild-type CAL-120 cells (**B**), and *PIK3CA* mutant SUM-159 cells (**C**), and *PIK3CA/PTEN* mutant CAL-51 cells (**D**) with S63845 (n=3 biological replicates). (ns = not significant).

Supplemental Figure 4

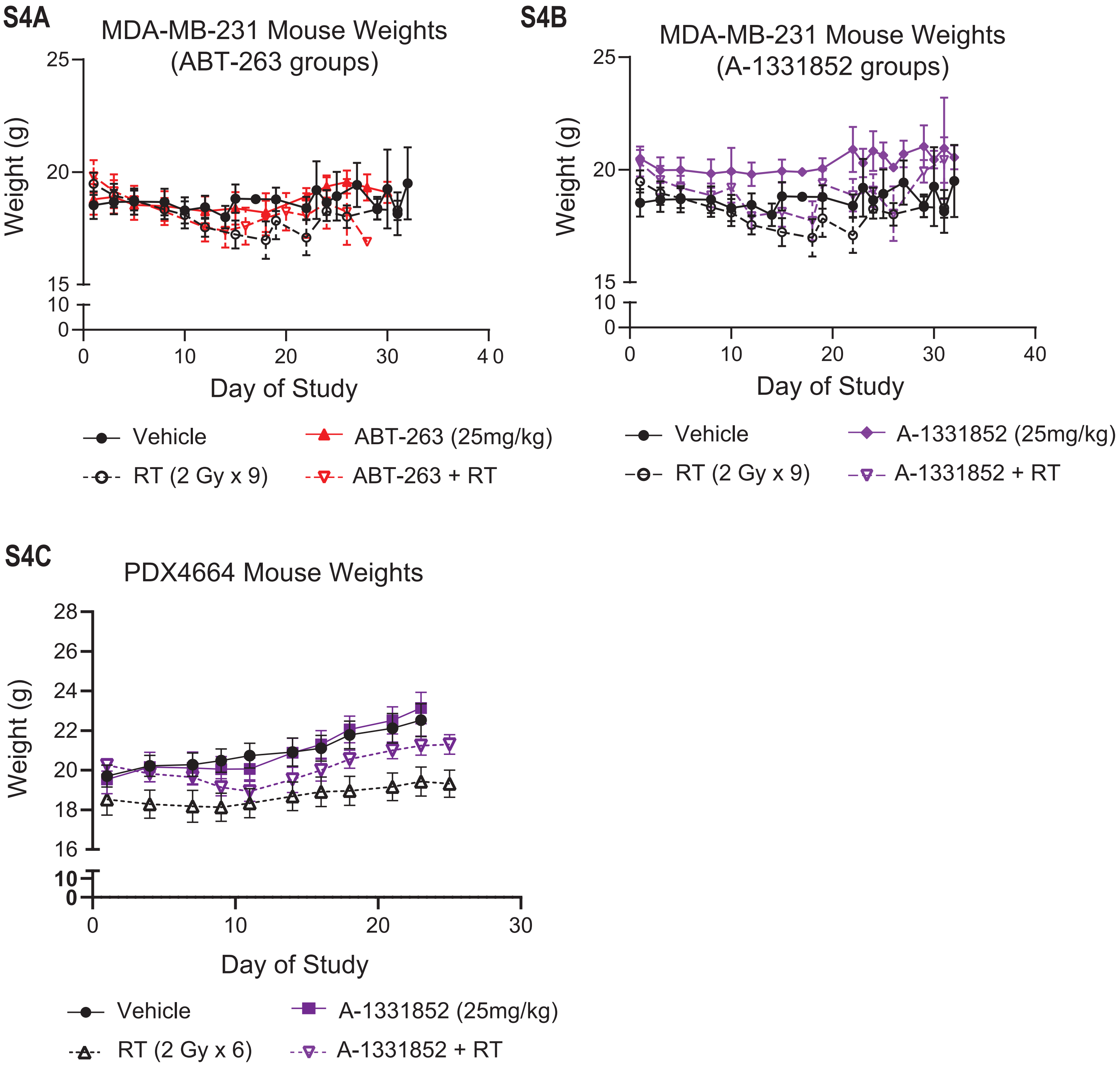

Supplemental Figure S4: **<u>Combination therapy does not lead to significant toxicities in vivo.</u>** Weights from mice bearing MDA-MB-231 xenografts were treated with ABT-263 (**A**) or A-1331852 (**B**) ± RT. The black/dashed curves for control and RT only groups represent identical data in **A/B** and are repeated on both graphs for visual clarity. Weights from mice bearing PDX4664 xenograft tumors were also measured throughout the duration of the study. Points represent the average of the of mouse weights (7-8 mice per group for MDA-MB-231 tumors and 6-7 mice per group for PDX4664) ± SEM.

Supplemental Figure 5

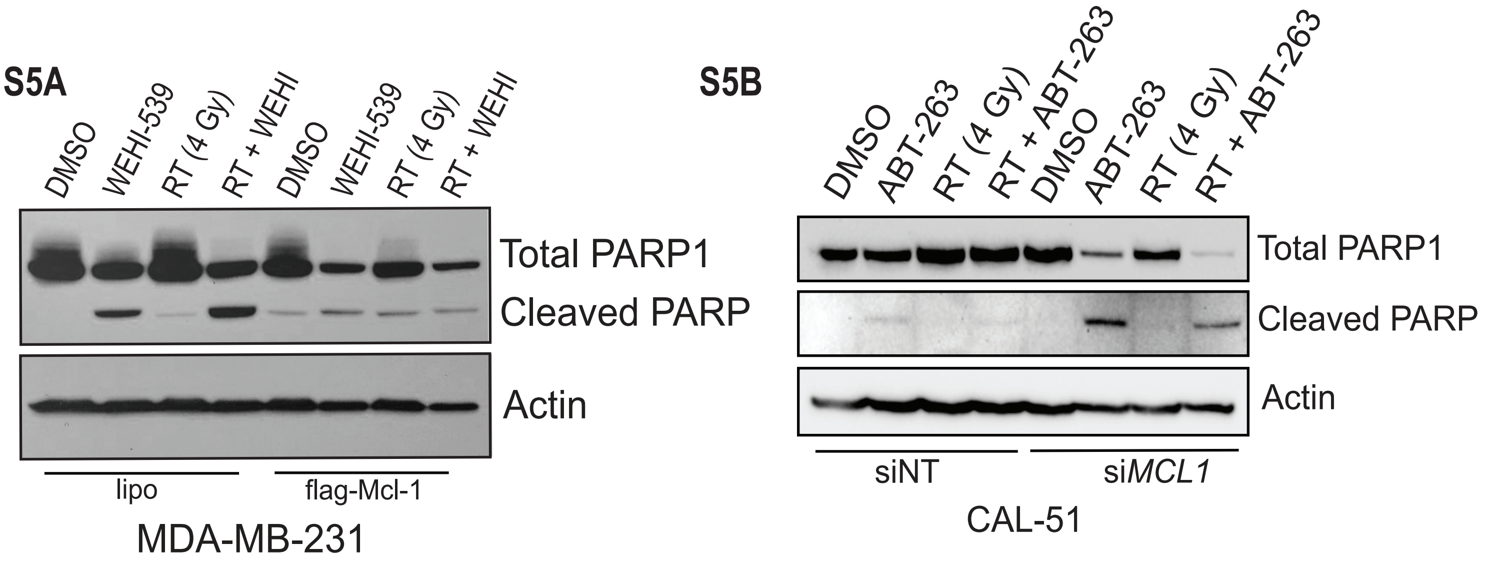

Supplemental Figure S5: <u>Manipulation of Mcl-1 modulates Bcl-xL-inhibitor mediated apoptosis.</u> Mcl-1 was overexpressed in MDA-MB-231 cells (**A**) or knocked down in CAL-51 cells (**B**); cells were pretreated for 1 hour before RT (4 Gy) and harvested 48 hours after RT to assess formation of cleaved PARP. Western blots are representative of duplicate biological experiments.

Supplemental Figure 6

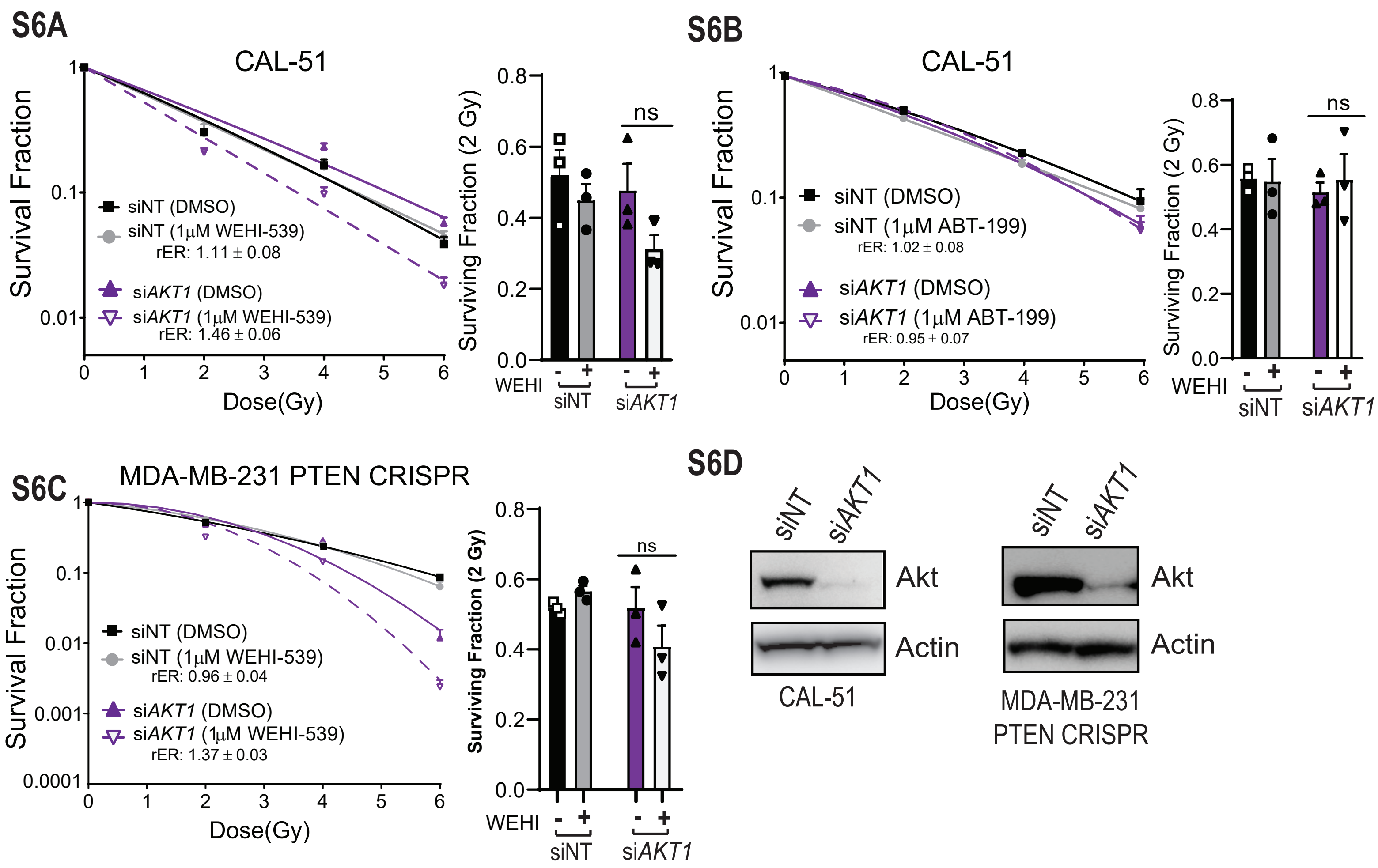

Supplemental Figure S6: <u>Akt is a modulator of Bcl-xL mediated radiosensitivity in TNBC cell lines.</u> Transient expression of *siAKT1* in CAL-51 (**A,B**) cells and MDA-MB-231 *PTEN* CRISPR (**C**) cells to perform clonogenic survival assays (n=3 biological replicates). Western blots were used to assess Akt expression following knockdown (24 hours post-transfection) (**D**). (ns = not significant, * = p < 0.05, ** = p < 0.01, *** = p < 0.001).

Supplemental Figure 7

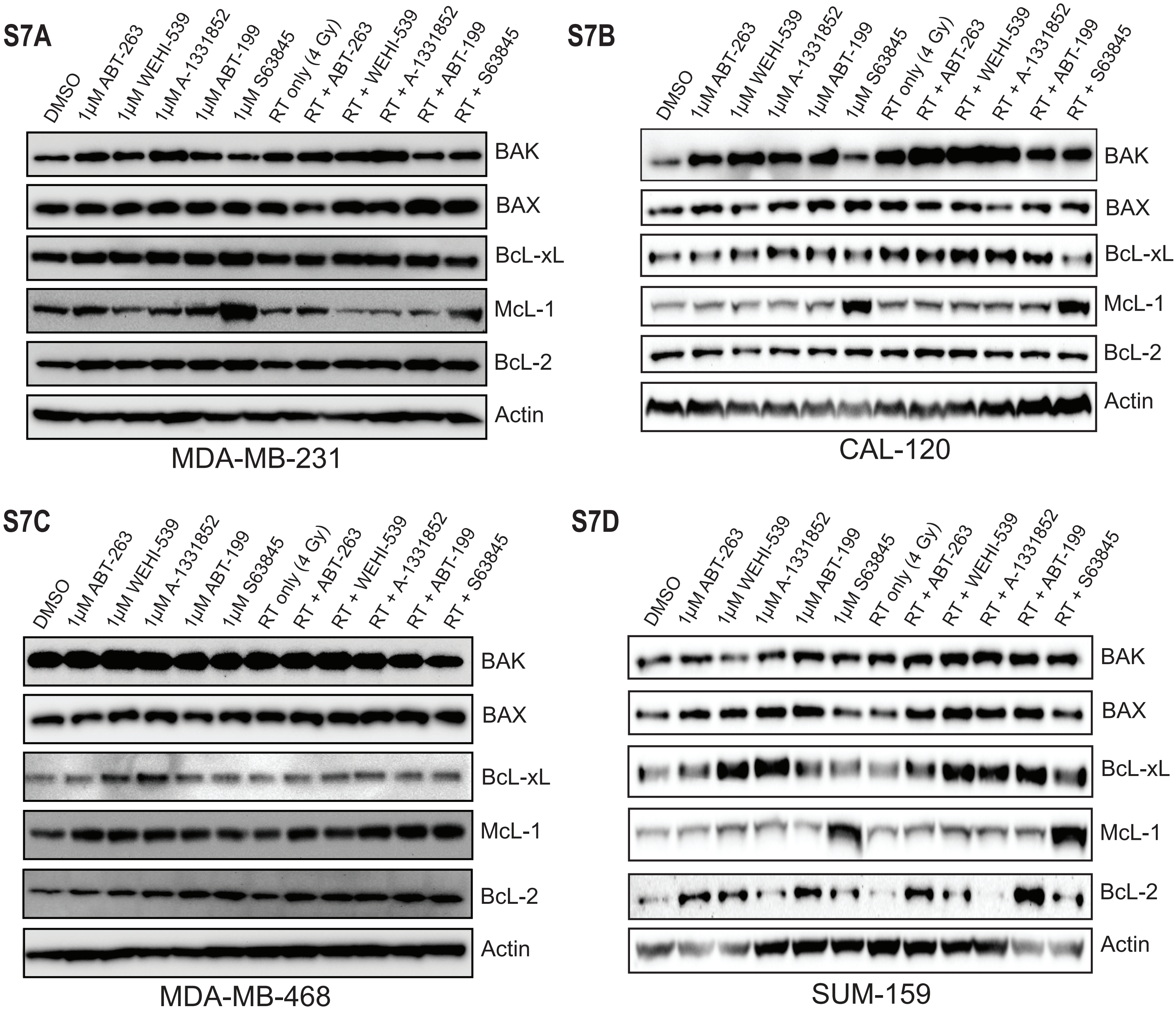

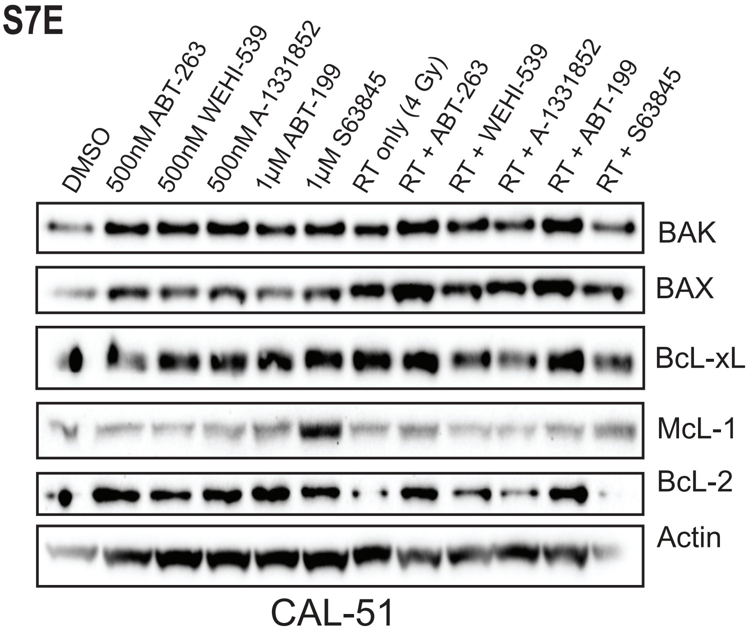

Supplemental Figure S7: <u>BAK, but not other mediators of apoptosis, is induced with radiosensitization in *PIK3CA/PTEN* wild type TNBC</u>. *PIK3CA/PTEN* wild type cell lines (**A-B**) or *PIK3CA/PTEN* mutant cell lines (**C-E**) were treated with drug (ABT-199, ABT-263, S63845, WEHI-539, A-1331852) one hour prior to RT (4 Gy) and cells/media were harvested 48 hours after RT. Blots are representative of duplicate biological experiments.

Supplemental Figure 8

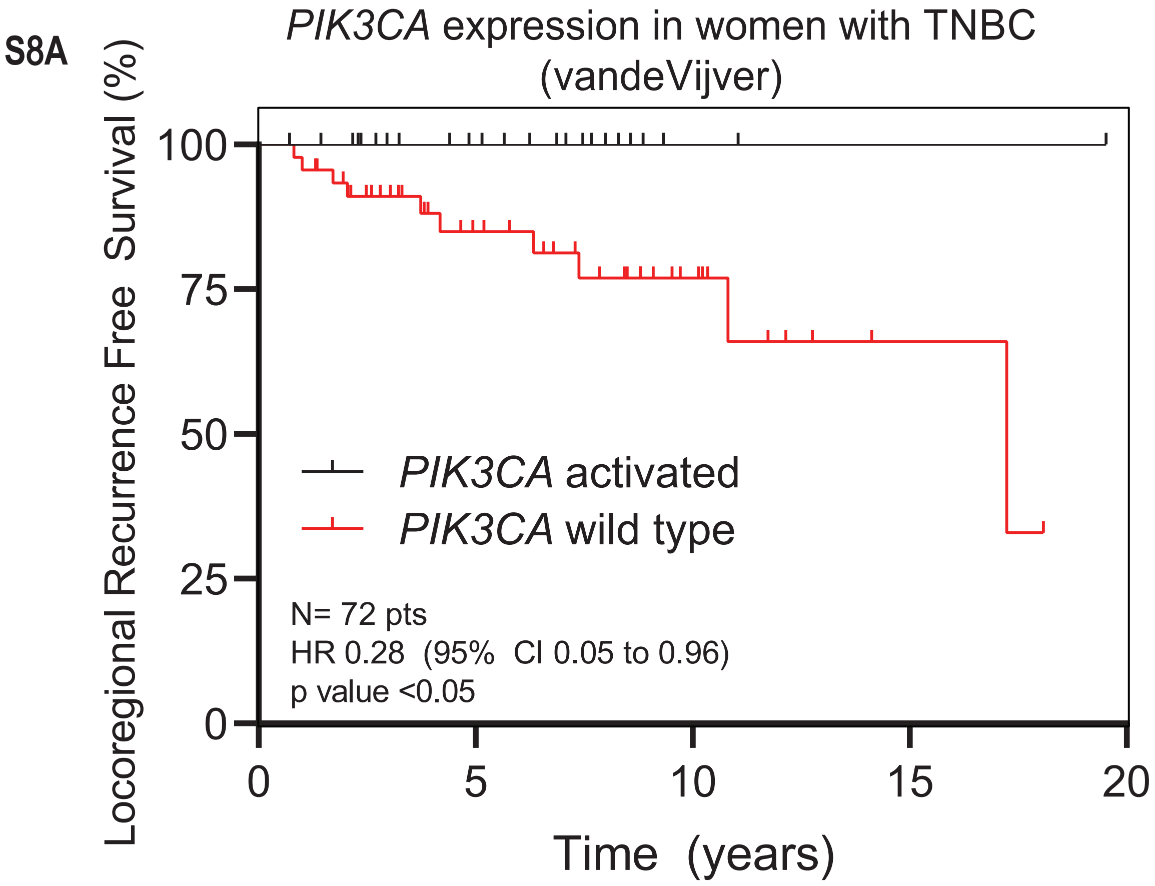

Supplemental Figure S8: Breast cancer recurrence rates are higher in women with TNBC and *PIK3CA* wild type tumors compared to mutant tumors. Kaplan–Meier recurrence free survival (RFS) and local RFS analysis in the vandeVijver dataset demonstrates that patients whose tumors have wild type *PIK3CA* have significantly higher rates of local recurrence after radiation than patients with activating PIK3CA mutations (**A**).

Supplemental Table 1

	PIK3CA	*PTEN*
**MDA-MB-231**	wt	wt
**CAL-120**	wt	wt
**CAL-51**	missense mutation (E542K)	Frameshift (E288, TK321)
**MDA-MB468**	wt	Spice Site / Frameshift (A72fsX5)
**SUM-159**	missense mutation (H1047L)	wt

Supplemental Table 2

Cell Line	IC_{50} ABT-263	IC_{50} WEHI-539	IC_{50} ABT-199	IC_{50} S63845	IC_{50} A-1331852
MDA-MB-231	1.3μM	1.2μM	> 5 μM	> 5 μM	940nM
CAL-120	610nM	158nM	> 5 μM	> 5 μM	20.5nM
CAL-51	> 5 μM	> 5 μM	> 5 μM	606nM	-
MDA-MB-468	> 5 μM	> 5 μM	> 5 μM	> 5 μM	-
SUM-159	4.28μM	> 5 μM	> 5 μM	4.8μM	-

Supplemental Table 3

MDA-MB-231 Fractional Tumor Volume (FTV) Calculations					
ABT-263			Combination		
Day	RT	ABT263	Expected	Observed	Ratio
8	0.914	0.801	0.732	0.761	**0.961**
12	0.770	0.716	0.551	0.702	**0.785**
19	0.553	0.581	0.321	0.407	**0.790**
29	0.428	0.485	0.208	0.269	**0.772**
Final	0.457	0.539	0.246	0.275	**0.895**

Supplemental Table 4

MDA-MB-231 Fractional Tumor Volume (FTV) Calculations					
A-1331852			Combination		
Day	RT	A133	Expected	Observed	Ratio
8	0.914	0.809	0.739	0.720	**1.027**
12	0.770	0.780	0.600	0.497	**1.207**
19	0.553	0.607	0.336	0.232	**1.447**
26	0.428	0.612	0.262	0.211	**1.244**
Final	0.457	0.719	0.328	0.234	**1.405**

Supplemental Table 5

PDX-4664 Fractional Tumor Volume (FTV) Calculations					
A-1331852			Combination		
Day	RT	A133	Expected	Observed	Ratio
7	0.694	0.813	0.564	0.645	**0.874**
14	0.492	0.815	0.401	0.362	**1.109**
18	0.420	0.812	0.341	0.286	**1.190**
21	0.376	0.842	0.317	0.249	**1.276**
Final	0.539	0.813	0.438	0.268	**1.638**

Supplemental Table S1: Triple negative breast cancer cell line *PIK3CA/PTEN* mutational status

Supplemental Table S2: IC_{50} values in TNBC cell lines with ABT-263, WEHI-539, and ABT-199.

Supplemental Table S3: Fractional tumor volume calculations for MDA-MB-231 xenografts treated with RT + ABT-263

Supplemental Table S4: Fractional tumor volume calculations for MDA-MB-231 xenografts treated with RT + A-1331852

Supplemental Table S5: Fractional tumor volume calculations for PDX4664 xenografts treated with RT + A-1331852
